# Predicting the unpredictable: critical analysis and practical implications of predictive anticipatory activity

**DOI:** 10.3389/fnhum.2014.00146

**Published:** 2014-03-25

**Authors:** Julia A. Mossbridge, Patrizio Tressoldi, Jessica Utts, John A. Ives, Dean Radin, Wayne B. Jonas

**Affiliations:** ^1^Department of Psychology, Northwestern UniversityEvanston, IL, USA; ^2^Dipartimento di Psicologia Generale, Universita di PadovaPadova, Italy; ^3^Department of Statistics, University of California at IrvineIrvine, CA, USA; ^4^Samueli InstituteAlexandria, VA, USA; ^5^Consciousness Research Laboratory, Institute of Noetic SciencesPetaluma, CA, USA

**Keywords:** presentiment, predictive coding, anticipatory activity, neural prediction, temporal processing

## Abstract

A recent meta-analysis of experiments from seven independent laboratories (*n* = 26) indicates that the human body can apparently detect randomly delivered stimuli occurring 1–10 s in the future (Mossbridge etal., 2012). The key observation in these studies is that human physiology appears to be able to distinguish between unpredictable dichotomous future stimuli, such as emotional vs. neutral images or sound vs. silence. This phenomenon has been called *presentiment* (as in “feeling the future”). In this paper we call it *predictive anticipatory activity* (PAA). The phenomenon is “predictive” because it can distinguish between upcoming stimuli; it is “anticipatory” because the physiological changes occur before a future event; and it is an “activity” because it involves changes in the cardiopulmonary, skin, and/or nervous systems. PAA is an unconscious phenomenon that seems to be a time-reversed reflection of the usual physiological response to a stimulus. It appears to resemble precognition (consciously knowing something is going to happen before it does), but PAA specifically refers to *unconscious* *physiological *reactions as opposed to *conscious premonitions*. Though it is possible that PAA underlies the conscious experience of precognition, experiments testing this idea have not produced clear results. The first part of this paper reviews the evidence for PAA and examines the two most difficult challenges for obtaining valid evidence for it: expectation bias and multiple analyses. The second part speculates on possible mechanisms and the theoretical implications of PAA for understanding physiology and consciousness. The third part examines potential practical applications.

## PART 1. THE EVIDENCE FOR PREDICTIVE ANTICIPATORY ACTIVITY

Predictive anticipatory activity (PAA) is defined here as statistically reliable differences between physiological measures recorded seconds before an unpredictable emotional event occurs vs. seconds before an unpredictable neutral event occurs. An emotional or arousing event is defined as an event that activates the sympathetic nervous system; while a neutral event activates the sympathetic nervous system to a lesser degree or not at all. A colloquial definition of PAA is “sensing the future,” or *presentiment* (e.g., Radin, 1997, 2004; Radin and Borges, 2009). Here we use the more descriptive term PAA to indicate that this phenomenon is *predictive* of randomly selected future events, *anticipates* these events more often than chance, and is based on *physiological* *activity *in the autonomic and central nervous systems. In this section, we present the evidence for this phenomenon. We then discuss its implications (Part 2) and potential applications (Part 3).

**FIGURE 1:**
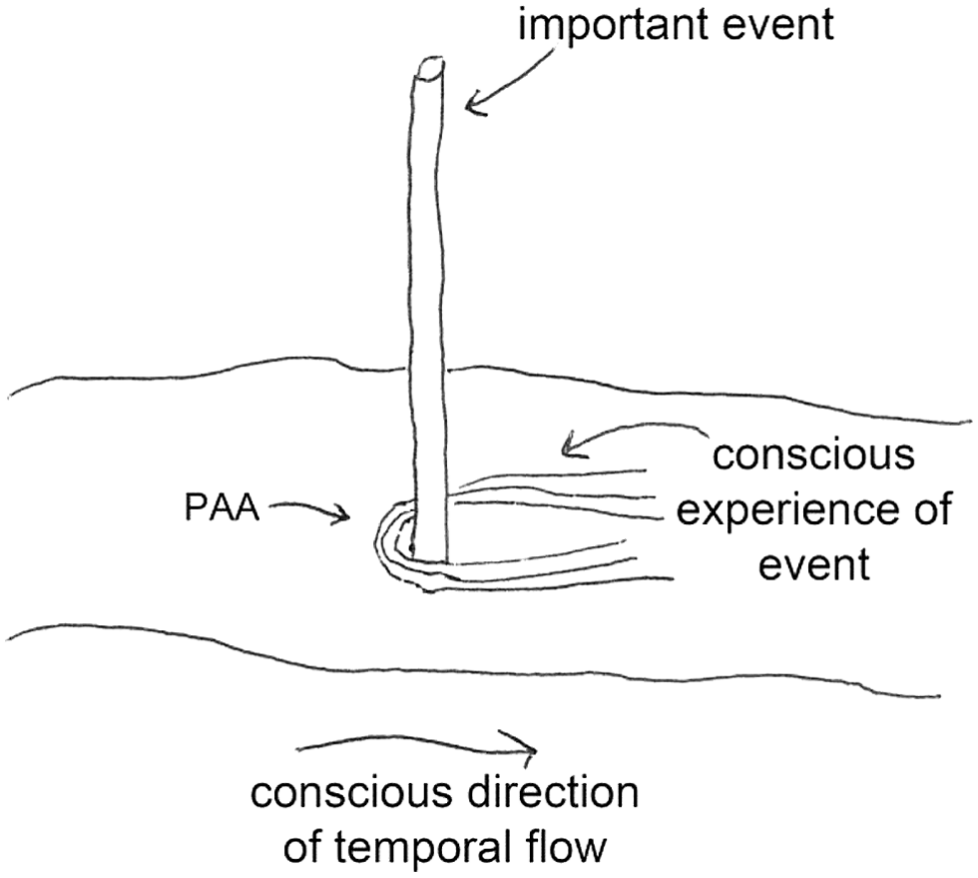
**Back-pressure perturbations from a downstream intrusion (an arousing/important event) in a stream of water may be a useful metaphor for predictive anticipatory activity (PAA).** We are not normally conscious of PAA effects because downstream perturbations are much larger in magnitude.

Predictive anticipatory activity is postulated to be an unconscious physiological phenomenon that may be thought of as a preview of our conscious awareness of future emotional or arousing events. A metaphor may help to provide an intuitive feel for this effect – watching a river move past a stick. The metaphor works as follows (**Figure 1**): imagine that the direction of the water’s current is the conscious experience of the flow of time (temporal flow), and imagine that an intrusion in the flow (the stick) is an emotional, arousing, or otherwise important event. The largest disturbance in the water made by the intrusion is downstream (in the “forward” time direction), which is analogous to our conscious reaction to experiencing the important event. But if one examines the flow of water near the stick, one will also see a small perturbation *upstream*, anticipating the intrusion in the water downstream due to the back pressure. Similar to PAA, this upstream perturbation is a hint of things to come. It is not normally part of our conscious awareness and, as with disturbances in a flow of water, the majority of the effect of an intrusion is downstream of the intrusion.

In contrast to PAA, *precognition* may be defined as a perception or a behavior (not a physiological measure) that is influenced by future events. For example, a recent series of experiments published in the *Journal of Personality and Social Psychology* suggested that perception of a future event may influence decisions and memory in the present (Bem, 2011; also see Maier et al., in press; Ritchie et al., 2012). Though it seems plausible that precognition is related to PAA, examination of that possibility is beyond the scope of this article.

**FIGURE 2:**
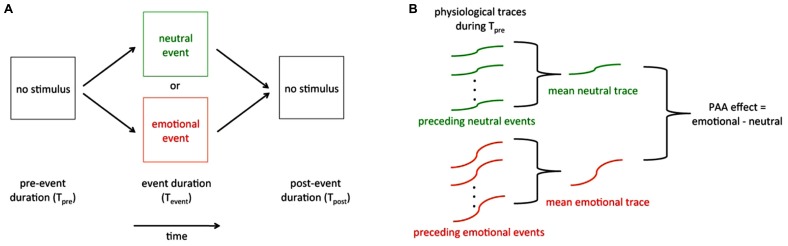
**Schematic of a generalized PAA trial and overall results. (A)** One trial of a series of trials. The pre-event (*T*_pre_), event (*T*_event_), and post-event (*T*_post_) durations are on the order of seconds. The physiological activities of interest are recorded continuously throughout the series of trials. **(B) **PAA effect. Data recorded during *T*_pre_ are baselined to a period preceding *T*_pre_ and are most often averaged across trials within each event type to provide an average *T*_pre_ response for the emotional event and an average *T*_pre_ response for the neutral event. The PAA effect is usually defined by the difference between these two averaged changes in *T*_pre_ physiology.

Experimental tests of PAA generally use one of two designs, both of which involve a series of randomly interspersed emotional and neutral events. The most common paradigm is one in which participants passively view and/or listen to a series of stimuli that are randomized in terms of stimulus type (e.g., emotional vs. neutral). A less common paradigm is one in which participants actively guess the outcome of each in a series of future events. In both paradigms, care must be taken to ensure that a truly random series of events is generated and that participants or experimenters cannot infer the upcoming event type through usual sensory means. Physiological data (skin conductance, heart rate, respiration rate, EEG activity, etc.) are recorded continuously during the experiment (**Figure 2A**) Each trial may be assessed on a trial-by-trial basis (see Recommendations for Designing Reliable PAA Sensing Tools, below), but more typically T*pre* is evaluated by averaging it across multiple trials of similar types (**Figure 2B**; e.g., emotional vs. neutral) in the series.

More than 40 experiments investigating PAA in humans have been published over the past 36 years (including: Hartwell, 1978; Radin et al., 1995, 2011; Bierman and Radin, 1997; Radin, 1997, 2004; Don et al., 1998; Bierman, 2000; Bierman and Scholte, 2002; McDonough et al., 2002; Spottiswoode and May, 2003; McCraty et al., 2004a,b; Sartori et al., 2004; May et al., 2005; Tressoldi et al., 2005, 2009, 2011; Radin and Borges, 2009; Bradley et al., 2011).

This literature prompted a meta-analytic examination of PAA to assess the combined evidence and repeatability of the phenomenon (Mossbridge et al., 2012). The meta-analysis tested the hypothesis that the difference between physiology preceding emotional and neutral events is in the same direction as the difference after those same events; in other words, it tested the hypothesis that the pre- and post-event physiological differences have the same sign (positive or negative). Using statistically conservative methods, the analysis revealed a small but highly statistically significant effect size in support of the hypothesis [fixed effect: overall ES = 0.21, 95% CI = 0.15–0.27, *z *= 6.9, *p *< 2.7 × 10^-12^; random effects: overall (weighted) ES = 0.21, 95% CI = 0.13–0.29, *z *= 5.3, *p *< 5.7 × 10^-8^]. Higher quality studies produced a larger overall effect size and greater level of significance, indicating that lack of quality was not responsible for the significant result (Mossbridge et al., 2012)^1^.

It is important to note that a meta-analysis is only as good as the data that it examines. Both questionable research practices (QRP) and physiological artifacts have the potential to produce results that mimic a PAA effect. If QRPs are sufficiently widespread, they could potentially be responsible for the highly significant meta-analytic results discussed here. Possible bias can be introduced by experimenter fraud, selective reporting, filter artifacts imposed on the raw data, multiple analyses, and anticipatory and order effects. These and other possible explanations for PAA have been critically examined and found to be lacking in explanatory power (Mossbridge et al., 2012). Here we briefly examine two of the more important criticisms of the evidence for PAA: multiple analyses and order effects, with a focus on expectation bias.

### CRITICISMS OF THE EVIDENCE FOR PAA

#### p-Hacking

One QRP that appears to be common throughout behavioral, social and medical research is to try to create a statistically significant effect using alternative analyses when the originally planned analysis did not find a significant effect (Simmons et al., 2011). If some of the PAA researchers used this technique to produce a significant PAA result, without noting the results as *post hoc*, then a meta-analysis based on those results would have an inflated outcome. This activity, dubbed “*p*-hacking” because it involves cutting through the data in many ways to obtain a *p-*value low enough to declare statistical significance, is a concern when determining the validity of any reported experimental phenomenon. The question is whether *p*-hacking can explain the significant meta-analytic results for PAA.

To answer this question the authors performed a meta-analysis on a subset of the data. This subset consisted of studies using electrodermal activity as the measure of interest, and the meta-analysis of these studies confirmed the presence of a highly significant overall effect (Mossbridge et al., 2012). Electrodermal activity measures have fewer parameters than EEG and fMRI data, so a researcher who wished to perform *p*-hacking would have few parameter choices and thus fewer chances to get a significant effect. One critical parameter in electrodermal studies is the duration of the pre-stimulus activity under examination (T*pre*).**Interestingly, within each publication and more often than not between publications, experimenters who performed multiple studies using electrodermal activity as a measure of interest consistently used the same T*pre *duration (most often 3 s before the stimulus), even when significant results were not found (see Anticipatory Period column in Appendix table A1; Mossbridge et al., 2012). This is not behavior that is consistent with *p*-hacking strategies.

Another feature of the same PAA meta-analysis was that it tested a hypothesis that differed from any explicit hypothesis stated by the authors of the original studies. That is, for the studies included in the meta-analysis, when a hypothesis was formally stated it was of the form that there would be a statistically reliable *difference* between the pre-event physiology for an emotional upcoming event as compared to another (relatively neutral) upcoming event. The direction of this difference was usually not predicted. However, the hypothesis of the PAA meta-analysis was that the direction of the pre-event difference would explicitly mirror the direction of the post-event difference (Mossbridge et al., 2012). In other words, if the direction of the pre-event difference in a physiological measure was the same as the direction of the post-event difference in the same physiological measure, the effect size for the study was given a positive sign (in support of the hypothesis), and if the direction of the pre- and post-event differences were not the same, then the effect size was given a negative sign (in contradiction to the hypothesis). Thus, even if researchers *p*-hacked to get a significant effect in an individual study, it would not necessarily be an effect in the same direction as that tested by the meta-analysis, and therefore it would not necessarily support the hypothesis of the meta-analysis. In fact, several studies examined in the meta-analysis did show effects in directions that opposed the hypothesis of the meta-analysis itself. In this way the meta-analysis was decoupled from one possible source of *p*-hacking. Nevertheless, the cumulative results remained highly significant.

From these analyses, it appears that *p*-hacking and other forms of unreported multiple analyses are not a compelling explanation for PAA. However, it is always possible that the evidence for PAA is influenced by more subtle variations in analyses. Until there is a gold standard experiment that is replicated across laboratories using exactly the same experimental procedure, physiological measures, and statistical analyses, there remains the possibility that multiple analyses could influence the body of evidence supporting PAA. Toward this end, we recommend that all researchers who investigate PAA register their experiments in advance, at any of several registries designed for experiments examining exceptional experiences^2^ or at a general experimental research registry^3^.

#### Order effects and expectation bias

Order effects may occur in any experiment with multiple sequential trials, including PAA studies. For example, *forward priming* describes a situation in which previous events influence responses to future events. Thus, responses to the word “flower” are faster if the word is preceded by the word “tree” vs. the word “knife” (Meyer and Schvaneveldt, 1971). Psychophysiologists who wish to avoid priming effects typically use randomization methods to help ensure that it is unlikely for a majority of the participants to systematically experience the same trial order. This increases the likelihood that spurious order effects will average out over participants and will not influence the cumulative results. In addition, the greater the number of trials in any given experiment, the less likely that similar trial orders may occur. If order effects are largely responsible for PAA, there should be a significant negative correlation between study effect size and the number of trials performed. However, this is not the case (Mossbridge et al., 2012).

While order effects do not appear to be a problem in these data, expectation bias is a more subtle effect that requires closer examination. Expectation bias is related to the human propensity to expect a “tail” in a coin toss after observing a series of “head” outcomes (the gambler’s fallacy). The reason expectation bias can potentially explain PAA is that a series of (randomly selected) neutral stimuli may produce a physiological shift toward excitement as the presumably imminent emotional trial approaches. In a sequence of trials with several such series of neutral events preceding emotional events, simulations suggest that the resulting physiological data could mimic a PAA effect (Dalkvist et al., 2002; Wackermann, 2002). Thus to understand the mechanisms underlying PAA, it is crucial to determine for each PAA experiment whether expectation bias was a potential explanation for the reported outcome.

There are several ways to quantify expectation bias. For example, one can examine a plot of the physiological measure of interest during T*pre* for an emotional event vs. the number of neutral trials preceding that emotional event. If expectation bias is a viable explanation for the PAA effect in a given experiment, then the activity during T*pre* for emotional events with greater numbers of neutral events preceding them will be larger than for those with fewer neutral events preceding them (Radin, 2004). Of the 26 studies examined in the recent PAA meta-analysis, 19 of them used this method or similar methods to empirically determine whether expectation bias could explain the PAA effect. None of them found that it could. Further, the overall effect size of the subset of studies that performed expectation bias analyses was greater than the effect size of the seven studies that did not perform such analyses, lending little support to the idea that expectation bias creates the PAA effect in general (Mossbridge et al., 2012). However, it is worth noting that studies revealing larger effects usually include more details about attempts to account for mundane explanations such as expectation bias, so there may be an inherent bias here.

Several statistical methods can be used to correct for expectation bias if evidence for that bias is found (e.g., Dalkvist et al., 2013). At least one of us (Mossbridge) has found an expectation bias effect in one PAA study, but because removing the bias produced a larger PAA effect, expectation was not a viable explanation. The bias was removed by discarding data from all but the first trial of the experimental session, because any significant PAA effect on the first trial could not be explained by expectations produced by preceding trials (Mossbridge et al., 2011). Importantly, this method revealed a larger PAA effect than the traditional trial-averaging method (compare Figures 1–6 in Mossbridge et al., 2012). This stronger effect could be due to the reduction of “temporal blurring” when physiological measures preceding only the first trial are examined (see Implications of PAA for Physiology and Consciousness Research, below). Based on this larger effect when only the first trial is considered, experiments are underway in which each individual performs only a single trial^4^. Despite obvious drawbacks due to the increase in inter-individual noise, and the effort involved in collecting data, this approach guarantees that expectation bias is not a viable explanation for any observed PAA effect.

The remainder of this article is based on the assumption that PAA reflects a true anticipatory prediction rather than being a physiological artifact or the result of bias and QRP. This assumption is made to allow us to explore the concept of PAA beyond the initial existential question.

### TYPES OF ANTICIPATORY PHYSIOLOGICAL ACTIVITY

Three broad categories of anticipatory physiological effects are well established in neuroscience and psychophysiology: anticipation of intentional motor activity, anticipation of stimulus detection, and anticipation of a complex firing pattern. PAA may be a novel, fourth category, or it may overlap with one or more of the three established categories.

Anticipation of intentional motor activity is supported by neurophysiological evidence indicating that the neural anticipation of our conscious awareness of having a will to move occurs at least 500 ms (Libet et al., 1983; Haggard and Eimer, 1999) and as much as 10 s (Soon et al., 2008; Bode et al., 2011) before the first conscious report of the will to move. Anticipation of stimulus detection is supported by the fact that EEG alpha activity during the pre-stimulus period for trials presenting stimuli that will be detected differs from alpha activity during pre-stimulus periods preceding stimuli that will not be detected (Ergenoglu et al., 2004; Mathewson et al., 2009; Panzeri et al., 2010). The explanation here is that specific phases and/or amplitudes of neural oscillations facilitate or suppress detection of the upcoming stimulus. Also, anticipation during sleep of a complex firing pattern to be used in the future, a phenomenon dubbed “preplay,” has been observed in mouse hippocampal neurons during sleep before entering a novel maze (Dragoi and Tonegawa, 2011). The firing pattern recorded during sleep has greater-than-expected similarity to the patterns recorded when the mouse eventually navigates the maze, an effect explained by the researchers with the idea that the hippocampus recycles generalizable firing patterns from its recent history to create the complex firing patterns accompanying spatial exploration. For these three categories of anticipatory effects, reasonable explanations using the usual cause-preceding-effect assumption are sufficient to explain the results. The usual causal temporal assumptions do not suffice, however, when attempting to understand PAA, because it apparently represents a statistically reliable *retrocausal* effect. In the next section we examine potential mechanism for PAA.

## PART 2. POTENTIAL MECHANISMS FOR PAA AND IMPLICATIONS OF PAA

### POTENTIAL MECHANISMS FOR PAA

#### Delayed conscious experience

One seemingly reasonable explanation for PAA is that our conscious mind is wrong about when events occur. That is, our conscious experience of events is delayed by seconds relative to some external/physical time of which we are not conscious. Meanwhile, unconscious neural processes are much less delayed relative to this external time. The explanation goes as follows: one important role of the unconscious is to assess the environment and mobilize physiological resources when it senses challenging external events. Once resources are mobilized and the body is readied, the conscious mind is presented with an ordered version of events that is necessarily temporally delayed so the conscious mind does not initiate counter-productive actions that might interfere with the preparation of physiological resources. Because challenging external events can occur at any time, the conscious mind is always receiving delayed and filtered information about sensory and motor events. Virtually all behavior is unconscious, and conscious awareness rides on top of this activity like an unfolding and delayed story.

This *delayed conscious experience* hypothesis predicts that we should find brain areas with activity that predicts upcoming consciously perceived events seconds before they occur. As mentioned previously, outside the PAA literature, Bode et al. (2011) and Soon et al. (2008) have reported that up to 10 s before the conscious experience of a decision to produce a motor event, brain activity predicts conscious decisions. These data support the delayed conscious experience hypothesis, but do they reflect PAA?

Although PAA may seem like a sensory counterpart to the predictive coding observed in the motor system, it differs in terms of the order of events and by not involving inferred events or making a decision. In PAA experiments, the physiological and stimulus events are in the wrong order to be explained causally, and they are time-stamped by a computer (not subjectively reported by research participants). Regardless of the absolute times when these events occur, a physiological reaction occurs before the stimulus to which it seems to be linked. Thus PAA neither supports nor refutes the delayed conscious experience hypothesis, but this hypothesis is not a viable explanation for PAA.

#### Quantum biology

A potentially more viable way to understand PAA effects is that they might reflect an epiphenomenon associated with quantum processing in biological systems. Aharonov et al. (1964, 1988) suggested that one way to explain quantum effects is via interactions between future and past events. This idea has recently been supported by advances in quantum measurement, so-called “weak measurements,” which demonstrate that observations in the future do indeed affect observations in the past (Aharonov et al., 1964, 1988; Hosten and Kwiat, 2008; Dixon et al., 2009). Further support of a similar “retrocausal” phenomenon in physics is provided by experimental verification of delayed-choice entanglement (Ma et al., 2012). Finally, because quantum effects have been shown to manifest in biological systems at physiological temperatures, e.g., in photosynthetic reactions (e.g., Scholes, 2011; Dawlaty et al., 2012; Olaya-Castro et al., 2012), it is no longer inconceivable that retrocausal quantum effects can occur in the human nervous system.

However, one problem with a quantum biological explanation for PAA is that retrocausal effects *on the order of seconds* would have to be explainable via quantum processes, and we know of no evidence so far that these effects can occur at that time scale^5^. Nevertheless, exploration into biological quantum effects is in its infancy, and most biological models have yet to entertain the consequences of retrocausation. Thus, the idea that PAA may be related to quantum effects is speculative and currently difficult to test. However, the quantum biology hypothesis demonstrates the value of anomalous phenomena in driving science forward by motivating scientists to search for novel explanations based on emerging scientific concepts. For further discussion of the philosophical and quantum mechanical arguments for time symmetry and retrocausation the reader is referred to an article on backward causation in the Stanford Encyclopedia of Philosophy^6^ and to Bierman (2010).

### IMPLICATIONS OF PAA FOR PHYSIOLOGY AND CONSCIOUSNESS RESEARCH

#### Physiology

The most mundane implication of PAA for physiologists is that the time-honored convention of establishing a baseline for physiological post-event measures by subtracting mean pre-event activity may obscure important physiological effects in two ways (Bierman and Radin, 1997; Mossbridge et al., 2012). First, by assuming that pre-event activity is equivalent between event types (without testing this assumption), PAA may be hiding in plain sight. Indeed, several re-examinations of pre-event activity reported in psychophysiology studies conducted for other purposes suggest that the PAA effect actually is present but overlooked (Bierman, 2000; Mossbridge et al., 2012). Of course, when researchers are performing a conventional psychophysiology experiment it is unlikely that they will feel the need to closely examine expectation bias or use truly random techniques to specifically rule out order bias. Thus PAA results found in data from such studies could potentially be due to these mundane explanations.

A second way that baselining data to pre-event physiological activity could obscure important physiological effects is by falsely increasing or decreasing post-event physiological differences. This can occur because pre-event activity is rarely equivalent between event types due to PAA, so subtracting these differing values can produce misleading post-event data as a result.

A more intriguing implication of PAA for those attempting to understand human physiology is that there seems to be a correlation between pre- and post-event responses, such that the magnitude of a post-event response seems to correlate with the magnitude of the corresponding pre-event response. Note that a simple correlation between pre- and post- responses is not what is being discussed here; certainly across individuals there is a strong correlation between the physiological state before and after any event, partially due to the fact that there are stronger inter-individual physiological variations than intra-individual variations. Instead, what has been observed is a correlation between the magnitude of the *change* in a physiological measure before an event and the magnitude of a *change* of that physiological measure after the event. We call this “temporal mirroring.” To investigate the idea of temporal mirroring in PAA, Radin (2004) quantitatively tested temporal mirroring using independent ratings of emotionality for different stimuli and found a significant correlation between pre-stimulus response size and stimulus emotionality. In another study, men had large blood-oxygen level dependent (BOLD) post-responses to erotic images randomly distributed along with violent and neutral images, and in men the only significant PAA effect occurred for erotic but not violent images (Bierman and Scholte, 2002). Meanwhile, women in the same study had large BOLD post-responses to violent but not erotic images, which also matched their PAA effects. Others have noted similar gender effects for which post-event differences in responding mirrored pre-event differences (McCraty et al., 2004b; Radin and Lobach, 2007; Radin and Borges, 2009; Mossbridge et al., 2011).

Another parameter dependence suggesting temporal mirroring is the relationship between PAA effects for single-modality events (auditory or visual stimuli presented alone) vs. events that are more ecologically valid in that they incorporate multiple modalities (e.g., emotional auditory and visual stimuli presented simultaneously). Well known in the multimodal literature is the idea that responses to stimuli presented in multiple modalities are more robust than responses to each modality alone (e.g., Meredith and Stein, 1986; Meredith et al., 1987). According to the “Hypothesis of Functional Equivalence,” any pre-stimulus PAA activity, if it exists, should be drawn on with same readiness as a post-stimulus response to a stimulus (Carpenter, 2012), supporting the functional value of temporal mirroring. Along these lines, in at least one report (Radin, 2004), PAA effects for events presented using multiple modalities are quantitatively larger than PAA effects for single-modality events, though several methodological differences between experiments preclude strong conclusions being drawn from these data.

Thus, it appears that the pre- and post-event physiological responses may mirror one another in size across participants (though pre-event responses are generally smaller than their post-event counterparts), implying that physiological processes are predicting either the importance of the future event to the organism or perhaps the future physiological response itself. These two interpretations may seem similar, but they have different implications for understanding the physiological mechanisms underlying PAA. The idea that PAA predicts the importance of the future event to the organism suggests that even if no physiological post-event response occurs due to some manipulation of the organism’s physiology or due to a probable event not occurring, PAA would occur before a highly probable important event. In contrast, if PAA predicts an organism’s future physiological response, then no PAA effect would occur if no physiological post-event response occurs. These differing interpretations of the pre-to-post-event mirroring phenomenon have important implications for applications that attempt to harness and amplify PAA, and these implications will be discussed below (see Part 3: Potential Applications of PAA Sensing Tools).

One final implication of PAA for our understanding of physiological systems is that PAA responses apparently decay with time prior to an event. If the size of a PAA response did not decay with time, one would never expect to find PAA, as arbitrarily timed future events that are important would be “temporally blurred” with arbitrarily timed future events that are not important. This is clearly not the case, as inter-trial intervals as short as 10 s have produced significant PAA effects. This does not necessarily mean that PAA effects completely vanish beyond 10 s, but it does indicate that there is some decay of PAA with time preceding the event. Thus, the physiological mechanisms underlying PAA are temporally localized in relation to each event^7^.

#### Consciousness

A major implication of PAA for our understanding of consciousness is that there must be a necessity for PAA to remain non-conscious most of the time. That is, for most people at most times there is a clear difference between the forward temporal flow of information and experience of which we are aware and a seemingly symmetric flow of information within the non-conscious portions of our experience, as evidenced by the existence of PAA. Why should this be the case? If some part of our nervous system can obtain information about events seconds in the future, would we not have evolved to make this information conscious?

One answer to this question is that the information is not conscious because most of the time it is not useful, like the majority of information that is processed unconsciously. Under this assumption, the mirroring of future physiological states by our unconscious physiological processes is just a side effect of how physiological systems (and in a more general sense the unfolding of events in time) work. The idea is that there has to be a physiological post-event response to produce the PAA prediction of that response, so there is then no point to being consciously aware of PAA effects as the event will occur in short order and there may be nothing we can do to stop it.

A seemingly paradoxical PAA experiment called a “bilking experiment” is one in which a participant’s PAA response is used to avoid a future emotional event that presumably caused the PAA response to occur in the first place. If such a PAA response can be shown to exist when there is no accompanying future emotional event, this would invalidate the idea that PAA requires a post-stimulus response and would support the idea that PAA predicts probable vs. actual events. One of us (Tressoldi et al., 2013) has preliminary data that support this idea. However, such bilking experiments are in their infancy, making it difficult to draw conclusions.

Another answer to “Why aren’t we conscious of PAA?” is that the conscious mind is not especially skilled at making quick decisions. Unconscious processing is increasingly being recognized as a powerful resource that provides the results of its calculations to conscious awareness for further use and elaboration (e.g., Kahneman, 2011). Converging evidence suggests that unconscious processing can result in learning and decision making that betters, or at least matches, those resulting from conscious processing (e.g., De Houwer et al., 1997; Dijksterhuis et al., 2006; Strick et al., 2011; Voss et al., 2012; Atas et al., 2013; Hassin, 2013). Thus, it might be evolutionarily advantageous for unconscious processing to assess upcoming events, filter them, mobilize resources, and only then inform conscious awareness (see *Potential Mechanisms for PAA *and* Delayed Conscious Experience*, above).

## PART 3. POTENTIAL APPLICATIONS OF PAA SENSING TOOLS

Assuming we can understand PAA well enough to amplify and characterize it for a given event of interest, the potential uses of PAA-sensing tools (PAASTs) largely depend on the delay between the PAA signal and the event of interest. Potential applications also depend on whether the PAA is caused by the high a priori probability of an emotional event or is the result of an actual and unavoidable emotional event.

Applications that could benefit from a few seconds advance notice may include: slamming on the brakes of a vehicle to prevent a crash, taking cover in advance of an explosion, or in general orchestrating quick movements to effect a fortuitous result within a few seconds. Applications requiring 10s of seconds might include: course corrections for vehicles, preparing to move out of a location, locating a hiding spot and then hiding there, preparing for a medical emergency, communicating information verbally, or in general, orchestrating more complex chains of action.

### POTENTIAL ROADBLOCKS TO DESIGNING RELIABLE PAA SENSING TOOLS

How does one amplify and characterize PAA for an event of interest so that one can determine the practical temporal window of usability for a PAAST? The amplification and characterization process itself could present several difficulties, which we speculate about here (see Recommendations for Designing Reliable PAA Sensing Tools, below, for potential solutions to these problems).

One critical problem to overcome during the characterization process is that when a PAAST is used outside of the laboratory, many events occur beyond the event of interest. For instance, one potentially life-saving application of PAASTs could be to predict the detonation of an improvised explosive device (IED). A PAAST device, when working perfectly, would emit an alert 10–20 s before the detonation of an IED, providing time to avoid the device or take cover. When working well, the PAAST device should not be triggered by emotional events such as an individual soldier’s phone call from a friend or a near miss in a firefight. The specific physiological PAA “signature” of a soldier to the IED detonation must be isolated.

When the signal is characterized and is being amplified, time course is likely critical. Any “temporal blurring” that might occur due to an overlap in physiological responses either before or after the IED detonation must be minimized. For example, if a soldier goes into a simulation where he will be faced with 20 IED detonations in a short time period, a temporal blurring of his or her physiology may occur because responses to later detonations will include responses to previous detonations. 

A third potential roadblock is that in the process of characterizing and amplifying PAA to a simulated IED detonation, the event itself may become uninteresting, producing small post-event physiological responses. If the post-event physiological response is small, this would likely reduce the size of the matching PAA response (see Physiology, above). Thus a soldier must be kept engaged and the simulated IED detonation must maintain its emotional and cognitive impact value.

**FIGURE 3:**
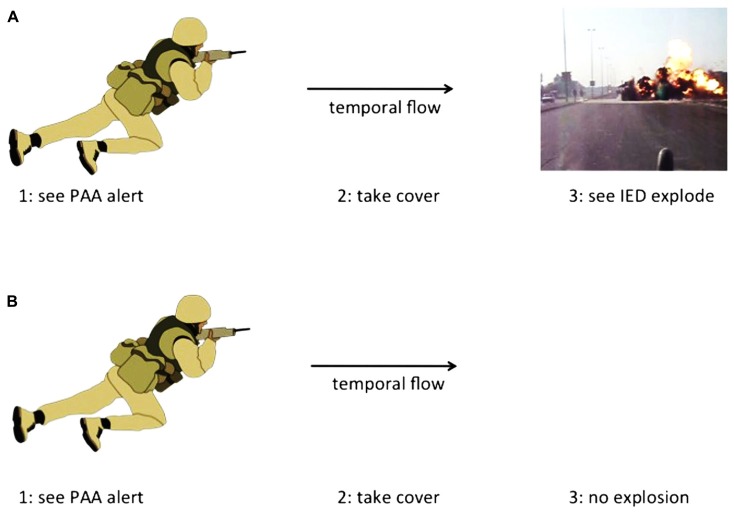
**Schematic illustrating two possibilities for the operation of a PAA-sensing tool. (A)** A soldier sees the PAA alert, takes cover, and sees the IED explosion from a safe location. **(B)** A soldier sees the PAA alert, takes cover, and does not see or hear the IED explosion. Note that in either case, injury was avoided, even though the second case seems theoretically paradoxical (see text for details).

Finally, it is important to address a perceived paradox that seems like a potential stumbling block, but may not be. The paradox goes like this: if PAA is a reflection of the future physiological state of an individual, and the actions taken once a PAA alert is given change the physiological state of that person, the PAA alert may not work in the first place. Imagine this scenario (**Figure 3A**): a PAAST gives a soldier an IED alert. The soldier takes cover away from a nearby trash heap but hears the sound of the subsequent explosion of the IED. The soldier has a typical post-detonation emotional response, which is what produced the PAA alert. Now imagine this paradoxical scenario (**Figure 3B**): a PAAST gives a soldier an IED alert. The soldier hides in a tank and puts on headphones. The soldier does not have a typical post-detonation emotional response, so there could not have been a PAA alert. However, there was a PAA alert. How could this work? If the second scenario can occur, it means one of two things: (1) that PAA does not reflect the future physiological state of the individual, or (2) that a non-typical post-detonation response is still an emotional response to saving one’s own life and so can still produce PAA. The PAAST still saved the soldier’s life, regardless of how it worked (see above for Potential Mechanisms for PAA and Implications of PAA). There may be a theoretical paradox here^8^, but perhaps not a practical one.

### RECOMMENDATIONS FOR DESIGNING RELIABLE PAA-SENSING TOOLS

Recommendations for designing reliable PAASTs are speculative, as they are based on results from studies varying broadly in their events of interests, methodology, and participants. However, here we make an attempt to outline best practices, as they would currently be defined, for designing a reliable PAAST.

The first step in designing any PAAST would seem to be finding the time course of the decay of PAA for the event of interest. Again, taking the example of IED detonation, once this delay is known, the optimal inter-detonation interval to produce the most reliable PAA effect will become apparent, and the usefulness of that delay can be assessed for the application. Thus, one critical initial experiment would be to use variations in the inter-detonation interval to determine the extent to which the PAA effect can be amplified by reducing temporal blurring with respect to the PAA response (see *Implications of PAA for Physiology and Consciousness Research* and* Physiology*, above).**

After finding the critical delay for the IED detonation event across a group of soldiers, the next step would be to characterize the specific physiological “tell” or signature of each soldier who will be using the PAAST. What are the respiration, temperature, blood volume, skin conductance, EEG, and heart rate signatures for each soldier? Obtaining this information would require exposing each soldier to multiple simulated experiences with IED detonations. Based on the little knowledge we have about PAA, these simulation protocols should share several characteristics. To ensure the robust emotional responses that produce reliable PAA, each simulated IED detonation should include at least auditory and visual information, with somatosensory and olfactory cues where possible. To reduce the influence of responses to previous events (temporal blurring) and to ensure continued strong responses after the upcoming event that seem to produce strong PAA (temporal mirroring), delays between simulated detonations should be relatively long (on the order of minutes or hours) and randomly timed. To ensure the generalizability of the PAA signature across multiple situations, detonations should occur in differing scenarios. Because the device must distinguish PAA to IED detonations from emotional responses to other events, events causing emotional responses (but that are not IED detonations) should be intermixed within the series of IED detonations. Finally, to ensure the continued autonomic responses to the IED detonation, any decrement in the post-stimulus response should be monitored and the simulation stopped at this point and resumed again when the post-stimulus response is robust.

During the entire simulation time, the soldier should be moving and behaving in a life-like environment while physiological data are being recorded continuously. Once enough simulated IED detonations are recorded (likely 30–60), the data from multiple physiological systems preceding each detonation can be analyzed on a trial-by-trial basis using non-linear machine-learning methods to find the characteristic PAA for that soldier. The reason we suggest automated learning is that non-linear, complex relationships between physiological variables and their time courses could exist that allow a fuller characterization of PAA. Along this line of thinking, one of us (Mossbridge) has preliminary data showing that such algorithms may be able to use EEG activity to determine the correct response to an upcoming random event (pressing the left or right mouse key as a correct response to an unpredictable event) on a trial-by-trial basis with above 75% accuracy in ¾ of untrained individuals^9^. We note that without the use of machine learning, finding the combination of electrodes and time points that would produce this predictive effect would have been difficult.

Characterizing the PAA for each soldier could be time consuming, and one possible way to reduce this investment is to screen soldiers to find those who have particularly reliable PAA effects, then characterize PAA to simulated IED explosions in only those soldiers. These PAA-sensitive soldiers could then use the resulting personalized PAAST as “a canary in the coal mine” for their whole team. Another possible time-saving method might be to run multiple soldiers on the IED simulation, then combine their data to characterize a generalized PAA to the simulated IED detonations that can be applied to soldiers who were not tested in the simulated environment. The reliability of this method, of course, would depend on the physiological similarity between the soldiers from whom the data were obtained and those in the field. One possible way to bridge this difference would be to use a generic PAAST on multiple soldiers in combat, so that, for instance, if three of four soldiers have their PAAST activated, all four soldiers would take cover. Assuming an algorithm combining the soldiers’ data would not alert the soldiers when only one soldier has a PAA response, this kind of generic physiological profile/multiple-user approach could potentially also reduce the chance of false alarms and increase the likelihood of avoiding real danger.

## SUMMARY AND CONCLUSIONS

In summary we have made the following points in this article.

• PAA, the predictive physiological anticipation of a truly randomly selected and thus unpredictable future event, has been under investigation for more than three decades, and a recent conservative meta-analysis suggests that the phenomenon is real.
• Neither QRP, expectation bias, nor physiological artifacts seem to be able to explain PAA.
• The mechanisms underlying PAA are not yet clear, but two viable yet difficult-to-test hypotheses are that quantum processes are involved in human physiology or that they reflect fundamental time symmetries inherent in the physical world.
• The evidence indicates that there is a temporal mirroring between pre- and post-event physiological events, so that the nature of the post-event physiological response is a reflection of the characteristics of the PAA for that event.
• Temporal blurring, in which closely overlapped emotional events may confuse or minimize both post-event responses and PAA before the event, may be a critical factor in isolating and amplifying PAA. %However, the noise introduced by this blurring may be limited by strictly “closing the temporal loop” between pre-stimulus and post-stimulus responses.
• The principles of temporal mirroring and temporal blurring both guide the recommendations for designing reliable PAASTs.
• Future research with multiple stimulus modalities, long inter-trial intervals, multiple individuals simultaneously exposed to the same stimulus, and machine-learning techniques will advance our understanding of the nature of PAA and allow a better harnessing of the delay before future events unfold.

## Conflict of Interest Statement

The authors declare that the research was conducted in the absence of any commercial or financial relationships that could be construed as a potential conflict of interest.

## Footnotes

1 Another meta-analysis using Bayesian statistics and including tests of PAA as well as tests for several other non-ordinary experiences revealed similar results (Tressoldi, 2011), but because that meta-analysis was not limited to PAA alone, it is beyond the scope of this review.

2

3 https://osf.io/

4 Dick Bierman has also performed a slightly different protocol of the single-trial study and found a presentiment effect, but this has not been published.

5 Elementary forms of coherence vanish quickly, but it is possible that coherence in living systems might be “pumped” and sustained for a relatively longer time.

6

7 The temporal issue might be related to the boundary conditions of each trial. In other words, the backward temporal information flow could end with the presentation of the stimulus. This could potentially be tested by delaying feedback over multiple trials, some of which are a long sequence of emotional targets, and some of which are a long sequence of calm targets.

8 If PAA predicts probable rather than actual futures, there would be no paradox.

9 For study results from the registered analysis, see
